# A new model to predict the influence of surface temperature on contact angle

**DOI:** 10.1038/s41598-018-24828-8

**Published:** 2018-04-25

**Authors:** Fabio Villa, Marco Marengo, Joël De Coninck

**Affiliations:** 1 University of Mons, Laboratory of Surface and Interfacial Physics (LPSI), 19 avenue Maistriau, 7000 Mons, BE Belgium; 2 University of Brighton, School of Computing, Engineering and Mathematics, Lewes Road, BN2 4GJ Brighton, UK

## Abstract

The measurement of the equilibrium contact angle (ECA) of a weakly evaporating sessile drop becomes very challenging when the temperatures are higher than ambient temperature. Since the ECA is a critical input parameter for numerical simulations of diabatic processes, it is relevant to know the variation of the ECA with the fluid and wall temperatures. Several research groups have studied the effect of temperature on ECA either experimentally, with direct measures, or numerically, using molecular dynamic simulations. However, there is some disagreement between the authors. In this paper two possible theoretical models are presented, describing how the ECA varies with the surface temperature. These two models (called Decreasing Trend Model and Unsymmetrical Trend Model, respectively) are compared with experimental measurements. Within the experimental errors, the equilibrium contact angle shows a decrease with increasing surface temperatures on the hydrophilic surface. Conversely the ECA appears approximately constant on hydrophobic surfaces for increasing wall temperatures. The two conclusions for practical applications for weakly evaporating conditions are that (i) the higher the ECA, the smaller is the effect of the surface temperature, (ii) a good evaluation of the decrease of the ECA with the surface temperature can be obtained by the proposed DTM approach.

## Introduction

The cohesive forces between molecules of the same phase (liquid, solid and gas/vapor) are responsible for the interphase tension of the liquid. The equilibrium balance of the forces acting on the liquid–vapor interface in contact with a solid surface determines the so-called “equilibrium contact angle” (ECA). At equilibrium, the ECA does not vary any more with time. The corresponding equation describing the balance of these forces is the well-known Young’s equation and it has been proved recently to be valid down to the nanometer scale^1,2^. The sum of forces parallel to the solid surface, per unit length of contact line, is perpendicular to the line and defines the local spreading coefficient γ_SV_−γ_SL_−γcosθ = γ(cosθ_Y_−cosθ), where θ is the measured local contact angle and θ_Y_ is the Young angle implied by the equation. Therefore, for a static condition, ideally the equilibrium contact angle θ_Y_ is equal to the measured θ. The local contact angle θ is a macroscopic quantity, with slight variation on the macroscopic scale, when the fluid surface is smooth. The value of this contact angle θ (CA) is affected by many parameters at very different length scales, for example the chemical properties of the surface, the surface roughness^3^ and the temperature of the liquid/vapour/solid system.

The correct value of this parameter at high temperature is critical in modelling and simulation of droplet evaporation^4,5^ and pool boiling^6,7^. In the numerical simulations, the wettability effect is normally introduced as a boundary condition at the wall in terms of ECA or the dynamic contact angle DCA^8,9^. The DCA depends on the contact line velocity and can strongly diverge with respect to the ECA^10,11^. The effect of surface wettability on bubble growth is normally incorporated in a Volume of Fluid (VOF) numerical model by imposing a prescribed ECA between the vapour/liquid interface and the heated solid surface^10^.

Several groups have studied the effect of surface temperature on the equilibrium contact angles either experimentally^12–16^, or even using molecular dynamic simulations^17–20^. However, there is still some disagreement between the authors about the dependency of the equilibrium contact angle on the system temperature for a given system pressure.

For Hydrophilic surfaces (HPiS in this work is defined as a surface with an equilibrium contact angle lower than 90°) the experiments show typically a reduction of the equilibrium contact angle with the increase of the surface temperature. In Aydar *et al*.^13^ contact angles of oils on polytetrafluoroethylene (PTFE) with different surface temperature (from 23 °C up to the oil smoke point at 200 °C) are measured and compared to values predicted by the Girifalco-Good-Fowkes-Young (GGFY) equation^21^. This equation, in combination with the Eötvös rule (that assumes an interface tension linear with the temperature), predicts a reduction of contact angle with increase of the surface temperature. Petke *et al*.^22^ measured the temperature dependence of contact angles of liquids on Hydrophilic and Hydrophobic (HPoS is a surface with an ECA greater than 90°) plastic surfaces, stating that the ECA decreases with the surface temperature in both cases. Not all the literature results agree with the reduction of the equilibrium contact angles with the surface temperature. Kenneth *et al*.^14^ examined surface temperature-dependence of the contact angle of water on graphite, silicon, and gold. The contact angle of water on various substrates did not monotonically decrease in every experiment, but also other behaviours have been observed. Kandlikar *et al*.^23^ measured the CA during rapid evaporation of liquid on a heated Hydrophobic surface and the values of the CA for high surface temperatures (100 °C < T_w_ < 250 °C) are remarkably constant. They showed that the liquid temperature has a much larger effect on the value of contact angles for polar liquids (water) than for apolar liquid (Diiodomethane), and for water they observed an increase of the CA with temperature, and a constancy of CA for diiodomethane. Another technique to study the effect of temperature on equilibrium contact angle is molecular dynamic (MD) simulation. What has been observed using MD is for HydroPhillic case a decrease of the ECA with temperature, and for Hydrophobic surface the contact angle increases with temperature. In^19^ Bruin *et al*. and also^24^ Blake *et al*. simulate a liquid-vapor interface which is confined by two parallel walls to study the contact angle versus the solid-fluid interaction strength. They found that the cos(θ) increases monotonically from −1 to 1 as solid-fluid interaction strength factor grows from 0.2 to 0.7. Outside this range the system gets unstable.

In the present work, two theoretical models (DTm and UTm) to predict the trend of the equilibrium contact angle (ECA) with temperatures are proposed. Equilibrium contact angle (called ECA) is defined in this paper as the time-average of the contact angle (called CA) in a given range (see eq. 3.31). Contact angle (CA) is the angle, conventionally measured through the liquid, where a liquid–vapor interface meets a solid surface, to a certain instant t. The experimental measurements of ECA of a sessile drop on heated surfaces are compared with these two theoretical models. The ECA is evaluated for sessile droplets at ambient pressure (1 bar) on surfaces with different wettabilities.

## Theoretical Models

Two theoretical models are proposed in order to estimate the ECA at different averaged temperatures:

- Decreasing trend model (DTm): this model provides a decreasing of the ECA with temperature for all type of surfaces (Hydrophobic and Hydrophilic).
- Unsymmetrical trend model (UTm): this model provides a decreasing of the ECA with temperature if the surface is Hydrophilic, and an increasing of ECA with temperature if the surface is Hydrophobic.

Moreover, in paragraph 2.4, a second law thermodynamic approach is used in order to bound the ECA behaviour for small temperature variations around the equilibrium condition of the three phases.

### Decreasing trend model – apolar liquid

The equilibrium contact angle reflects the balance between the relative strength of the liquid, solid, and vapor molecular interaction. The shape of a liquid–vapor interface is determined by the Young–Laplace equation, describing the equilibrium between solid–vapor interfacial energy (γ_sv_) the solid–liquid interfacial energy (γ_sl_) and the liquid–vapor interfacial energy (the surface tension γ_lv_). Fowkes^25,26^ has suggested a simple equation to describe γ_sl_ as function of γ_sv_ and γ_lv_ (the expression is only valid for substances interacting with additive dispersive forces and without hydrogen bonds, called apolar liquid):

2.1 $${\gamma }_{sl}={\gamma }_{sv}+{\gamma }_{lv}-2\sqrt{{\gamma }_{sv}{\gamma }_{lv}}$$

Using the expression in eq. 2.1, it is possible to eliminate γ_sl_ the write the into Young–Laplace equation without the solid–liquid interfacial energy γ_ls_ we get:

2.2 $$cos(\theta )=\frac{{\gamma }_{sv}-{\gamma }_{sl}}{{\gamma }_{lv}}=\frac{{\gamma }_{sv}-{\gamma }_{sv}-{\gamma }_{lv}+2\sqrt{{\gamma }_{sv}{\gamma }_{lv}}}{{\gamma }_{lv}}$$

Finally, the basic equation of DTm model is:

2.3 $$cos(\theta )=-\,1+2\frac{\sqrt{{\gamma }_{{sv}}}}{\sqrt{{\gamma }_{{lv}}}}$$

It is possible to note from eq. 2.3 that DTm requires two parameters to estimate the equilibrium contact angle: γ_sv_, γ_lv_. The γ_lv_ value at different temperatures can be found in^27^. Experimentally the surface tension decreases with temperature^28^. In the temperature range interesting for this work (20 °C < T < 150 °C) the function is generally considered linear with temperature for most of the fluids, and it assumes the form:

2.4 $${\gamma }_{lv}={\gamma }_{LV,0}(1-aT)$$

where *γ*_Lv,0_ is the surface tension of the liquid [Nm] at a reference temperature T_0_ (normally T_0_ = 20 °C) and a is the temperature coefficient [Nm/T]. The second value γ_sv_ is the surface tension of the solid. The parameter a is positive for one phase substance. If we suppose that the variation of the γ_sv_ in the tested temperature range (20 °C < T < 150 °C) is negligible compared to the variation of γ_lv_ in the same range^29^.

25 $${{\frac{\partial ({\gamma }_{LV})}{\partial T}|}_{{20}^{^\circ }{\rm{C}} < T < {150}^{^\circ }{\rm{C}}}\gg \frac{\partial ({\gamma }_{SV})}{\partial T}|}_{{20}^{^\circ }{\rm{C}} < T < {150}^{^\circ }{\rm{C}}}\approx 0$$

The experimental value of the equilibrium contact angle at ambient temperature (T = 20 °C) is used to extrapolate γ_sv_ for the tested surfaces, applying the following equation:

2.6 $${\gamma }_{sv}=\,cos\,{(\frac{{\theta }_{0}}{2})}^{4}({\gamma }_{LV,0}(1-a{T}_{0}))$$

### Decreasing trend model – polar liquid

The decreasing trend model can be extended for liquids with a-scalar forces (the combined polar interactions: dipole, induction, and hydrogen bonding) such as water. In this case it is necessary to use a different equation to describe γ_sl_ as function of γ_sv_ and γ_lv_. Owens and Wendt^30^ extended the formulation of Fowkes^25,26^ introducing the dispersion forces (van der Waals interaction) and a-scalar forces for the combined polar forces (e.g. dipole-dipole interactions and hydrogen bonding):

2.7 $${\gamma }_{sl}={\gamma }_{sv}+{\gamma }_{lv}-2\sqrt{{{\gamma }_{sv}}^{D}{{\gamma }_{lv}}^{D}}-2\sqrt{{{\gamma }_{sv}}^{P}{{\gamma }_{lv}}^{P}}$$

The total free energy at the surface is the sum of all the contributions, the dispersion and the polar intermolecular forces at the surface^26^:

2.8 $${\gamma }_{lv}={{\gamma }_{lv}}^{D}+{{\gamma }_{lv}}^{P}$$

D refer to the dispersion forces (van der Waals interaction) and A refer to the combined polar forces (e.g. dipole-dipole interactions and hydrogen bonding). Using eq. 2.7, it is possible to rewrite the Young–Laplace equation:

2.9 $$cos(\theta )=\frac{{\gamma }_{sv}-{\gamma }_{sl}}{{\gamma }_{lv}}=\frac{{\gamma }_{sv}-{\gamma }_{sv}-{\gamma }_{lv}+2\sqrt{{{\gamma }_{sv}}^{D}{{\gamma }_{lv}}^{D}}+2\sqrt{{{\gamma }_{sv}}^{P}{{\gamma }_{lv}}^{P}}}{{\gamma }_{lv}}$$

2.10 $$cos(\theta )=-\,1+\frac{2}{\sqrt{{\gamma }_{lv}}}\sqrt{{{\gamma }_{sv}}^{D}\frac{{{\gamma }_{lv}}^{D}}{{\gamma }_{lv}}+{{\gamma }_{sv}}^{P}\frac{{{\gamma }_{lv}}^{P}}{{\gamma }_{lv}}}$$

As already done in the apolar case, it is supposed that the variation of the γ_sv_ in the tested temperature range (20 °C < T < 90 °C) is negligible compared to the variation of γ_lv_ in the same range^29^.

211 $${{\frac{\partial ({\gamma }_{lv})}{\partial T}|}_{{20}^{^\circ }{\rm{C}} < T < {90}^{^\circ }{\rm{C}}}\gg \frac{\partial ({{\gamma }_{sv}}^{P}+{{\gamma }_{SV}}^{D})}{\partial T}|}_{{20}^{^\circ }{\rm{C}} < T < {90}^{^\circ }{\rm{C}}}$$

*γ*_*lv*_ is a linear function in T. It is supposed that both components are linear function of fluid temperature and by definition:

2.12 $${\gamma }_{lv,0}(1-aT)={{\gamma }_{lv,0}}^{D}(1-{a}^{D}T)+{{\gamma }_{lv,0}}^{P}(1-{a}^{P}T)\,\forall \,T$$

The temperature coefficients a^D^ and a^P^ are positive for one component liquid. It is possible to write the following inequality:

2.13 $$|{a}^{P}|\le |{a}^{D}|\le a$$

Therefore the ratio in eq. 2.10 can be considered constant in the range 20 °C < T < 90 °C and a polar liquid. The variation of the ratio in the temperature range is less than 1%:

214 $${{\frac{\partial (\frac{{{\gamma }_{lv}}^{P}}{{\gamma }_{lv}})}{\partial T}|}_{{20}^{^\circ }{\rm{C}} < T < {90}^{^\circ }{\rm{C}}}\le \frac{\partial (\frac{{{\gamma }_{lv}}^{D}}{{\gamma }_{lv}})}{\partial T}|}_{{20}^{^\circ }{\rm{C}} < T < {90}^{^\circ }{\rm{C}}}\mp \,1 \% \,\,{\rm{for}}\,{\rm{water}}\,{\rm{if}}\,0\le |{a}^{P}|\le |{a}^{D}|\le a$$

Therefore the term ${\rm{c}}={{\gamma }_{sv}}^{D}\frac{{{\gamma }_{lv}}^{D}}{{\gamma }_{lv}}+{{\gamma }_{sv}}^{P}\frac{{{\gamma }_{lv}}^{P}}{{\gamma }_{lv}}$ can be considered constant (with temperature) for water in the range 20 °C < T < 90 °C:

2.15 $$cos(\theta )=-\,1+\frac{2}{\sqrt{{\gamma }_{lv}}}\sqrt{c}$$

The experimental value of the equilibrium contact angle at ambient temperature (T = 20 °C) is used to extrapolate the constant parameter c:

2.16 $$c={(\frac{(cos({\theta }_{0})+1)\sqrt{{\gamma }_{LV,0}(1-a{T}_{0})}}{2})}^{2}$$

### Unsymmetrical Trend Model

In the Unsymmetrical Trend Model it is assumed that the difference of the interphase energy solid-vapor and solid-liquid $({\gamma }_{SV}-{\gamma }_{SL})$ is a weak function of the liquid temperature compared to *γ*_*LV*_:

2.17 $$cos(\theta )=\frac{{\gamma }_{sv}-{\gamma }_{sl}}{{\gamma }_{lv}}=\frac{const}{{\gamma }_{lv}}$$

This assumption means that $\partial ({{\rm{\gamma }}}_{{\rm{SV}}}-{{\rm{\gamma }}}_{{\rm{SL}}})/\partial {\rm{T}}=0$, in other words:

218 $${\frac{\partial ({\gamma }_{SV})}{\partial T}|}_{{20}^{^\circ }{\rm{C}} < T < {90}^{^\circ }{\rm{C}}}\approx {\frac{\partial ({\gamma }_{SL})}{\partial T}|}_{{20}^{^\circ }{\rm{C}} < T < {90}^{^\circ }{\rm{C}}}$$

The value of the equilibrium contact angle at ambient temperature (T = 20 °C) is again used to extrapolate the value of ${({\rm{\gamma }}}_{{\rm{SV}}}-{{\rm{\gamma }}}_{{\rm{SL}}})$ for the tested surfaces, applying the following equation:

2.19 $$({\gamma }_{SV}-{\gamma }_{SL})=\,cos({\theta }_{0})({\gamma }_{LV,0}(1-a{T}_{0}))$$

The value $({{\rm{\gamma }}}_{{\rm{SV}}}-{{\rm{\gamma }}}_{{\rm{SL}}})$ will be clearly positive for Hydrophilic surfaces $({{\rm{\theta }}}_{0} < 90^\circ )$ and negative for Hydrophobic surfaces $({{\rm{\theta }}}_{0} > 90^\circ )$ at fixed temperature T_0_.

### Thermodynamic approach

The influence of the temperature on equilibrium contact angle can be complementary approached using thermodynamics variables. In this approach, the Helmholtz free energy can be used to rewrite the surface tension:

2.20 $${\gamma }_{SV}-{\gamma }_{SL}={\rm{\Delta }}E-T{\rm{\Delta }}S$$

the Young–Laplace equation can thus be re-written as follow:

2.21 $$cos(\theta )=\frac{{\gamma }_{SV}-{\gamma }_{SL}}{{\gamma }_{LV}}=\frac{{\rm{\Delta }}E-T{\rm{\Delta }}S}{{\gamma }_{LV,0}(1-aT)}$$

Referring to a reference value of the temperature T_0_ which is connected to an equilibrium contact angle ${{\rm{\theta }}}_{0}$, the variation of the cos(θ) with respect to the reference value cos(θ_0_) is:

2.22 $$cos(\theta )-\,cos({\theta }_{0})=\frac{{\rm{\Delta }}E-T{\rm{\Delta }}S}{{\gamma }_{LV,0}(1-aT)}-\frac{{\rm{\Delta }}{E}_{0}-{T}_{0}{\rm{\Delta }}{S}_{0}}{{\gamma }_{LV,0}}$$

2.23 $$cos(\theta )-\,cos({\theta }_{0})=\frac{{\rm{\Delta }}{E}_{0}-{T}_{0}{\rm{\Delta }}{S}_{0}}{{\gamma }_{LV,0}}[\frac{\frac{{\rm{\Delta }}E-T{\rm{\Delta }}S}{{\rm{\Delta }}{E}_{0}-{T}_{0}{\rm{\Delta }}{S}_{0}}}{(1-aT)}-1]$$

2.24 $$cos(\theta )-\,cos({\theta }_{0})=\,cos({\theta }_{0})[\frac{\frac{{\rm{\Delta }}E-T{\rm{\Delta }}S}{{\rm{\Delta }}{E}_{0}-{T}_{0}{\rm{\Delta }}{S}_{0}}}{(1-aT)}-1]$$

For a Hydrophobic surface $({{\rm{\theta }}}_{0}\ge 90^\circ )$ $cos({{\rm{\theta }}}_{0})\le 0$. Suppose, according to the unsymmetrical trend model, that ${\rm{\theta }} > {{\rm{\theta }}}_{0}$ due to the temperature increases (T > T_0_):

2.25 $$cos(\theta )-\,cos({\theta }_{0}) < 0$$

Hence:

2.26 $$\frac{\frac{{\rm{\Delta }}E-T{\rm{\Delta }}S}{{\rm{\Delta }}{E}_{0}-{T}_{0}{\rm{\Delta }}{S}_{0}}}{(1-aT)} > 1$$

Considering $${\rm{\Delta }}S\approx {{\rm{\Delta }}S}_{0}$$

2.27 $$(T-{T}_{0}){\rm{\Delta }}{S}_{0}-({\rm{\Delta }}E-{\rm{\Delta }}{E}_{0}) < aT({\rm{\Delta }}{E}_{0}-{T}_{0}{\rm{\Delta }}{S}_{0})$$

The internal energy E is a monotonic function of the temperature. This relation shows a potential violation of the II principle if we assume an increase of the contact angle with temperature. Indeed Eq. 2.27 can be written as:

2.28 $$(T-{T}_{0}+aT{T}_{0}){\rm{\Delta }}{S}_{0} < k(T-{T}_{0}+aT{T}_{0})$$

where ${\rm{k}}={\rm{\Delta }}E/{\rm{T}}$. Eq. 2.28 is valid only if ${{\rm{\Delta }}S}_{0} < {\rm{k}}$ (ΔS_0_ and k are positive). But for a free energy:

2.29 $${\rm{\Delta }}{E}_{0}-{T}_{0}{\rm{\Delta }}{S}_{0}={T}_{0}(k-{\rm{\Delta }}{S}_{0}) > 0$$

which is violating the second principle. The thermodynamic approach shows that, if the temperature of all the three phases (solid, liquid and vapour) is the same, an increase of this common temperature state cannot induce an increase of the equilibrium contact angle. In many experiments, the three phases are not at the same temperature, for example the wall surface is kept at a higher temperature with respect to the liquid or vapour phase. In this sense the Unsymmetrical Trend Model can be still considered valid for a comparison with experimental results.

## Methods

**Figure 1:**
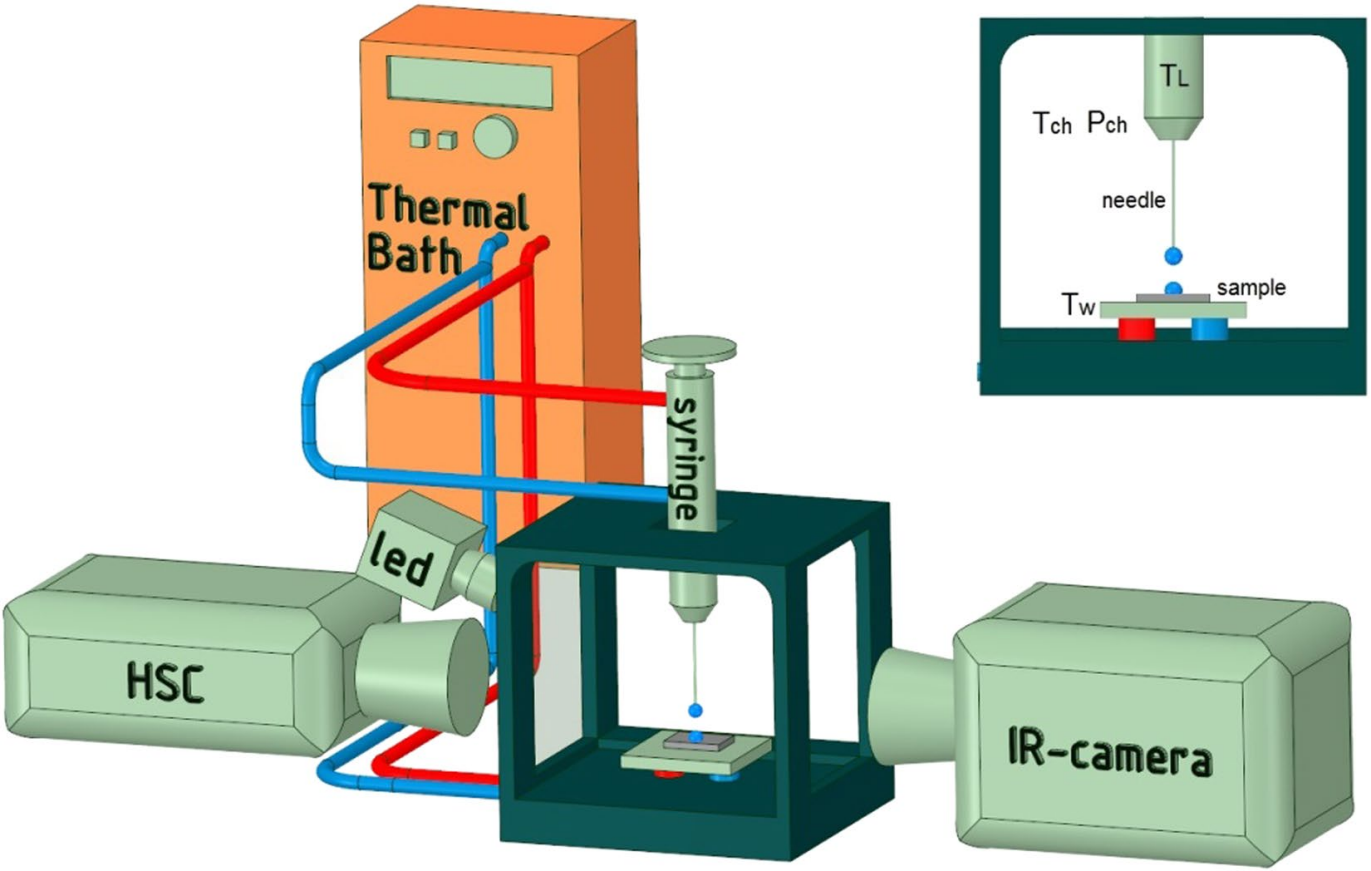
Experimental apparatus to measure the equilibrium contact angle. The system includes a drop shape analyser (DSA100-Kruss©), a thermal bath to change the temperature (Julabo© EC-5) and a IR-camera (SC5000 FLIR®).

The apparatus used in this work to measure the equilibrium contact angle is schematically described in Fig. 1. A drop shape analyser (DSA100-Kruss©) is used to measure the contact angle of a sessile drop on the selected surface. The sample and the syringe are connected to a thermal bath (Julabo© EC-5) in order to change the temperature of the system. An InfraRed-camera (SC5000 Flir®) is placed on the other side of the chamber to measure the average temperature of the droplet for some preliminary tests.

Table 1 reports the equilibrium contact angle (measured at ambient temperature 20 °C and P = 1 atm) for the tested surfaces. Water and Diiodomethane are the liquids used in these experiments. Both liquid have a surface tension greater than 50 mN/m, which affords high value even at high temperature. In the measurement of the ECA, the experiment is conducted in an environment with 3 species (solid, liquid and air-vapor) at ambient condition (P_ch_ = 1 atm and T_ch_ = 20 °C). The presence of air and the no saturation condition of the gas phase (vapor and air) can induce inaccuracy on the equilibrium contact angle measurement, as reported by Weisensee *et al*.^31^. A specific procedure to evaluate the ECA at medium high temperature is proposed in this paper. Four surfaces are tested: (a) politetrafluoroetilene (PTFE) and (b) polished aluminium samples, (c) SHS is a glass sample covered by a uniform and thin coating to generate super-Hydrophobicity, it is using a commercial product (Glaco Mirror Coat Zero©), (d) NP is a glass sample covered by a film of nanoparticles (silicon dioxide with polydimethylsiloxy group). Table 2 shows the physical properties for the two tested liquids (water and diidomethane).

**Table 1:** ECA of the surfaces, at temperature T_0_ = 20 °C, measured with sessile drop method. The mean surface roughness (R_a_) is also reported.

Surface	liquid	ECA [°]	Ra [µm]
Aluminium	water	68.6 ± 5.0	0.135
PTFE	water	110.6 ± 2.2	0.188
SHS	water	148.6 ± 3.6	0.045
NP	diiodomethane	152.6 ± 3.4	0.07

**Table 2:** Physical properties of the liquids. T_sat_ is the boiling temperature (at 1 atm).

	Water	Diiodomethane
T_sat_ [°C] −1 atm	100	181
Density [kg/m^3^]	973	3300
Dynamic viscosity [mPa s]	0.283	2.76
Total free energy of the liquid (*γ*_*LV*_) [mN/m]	72.10	50.88
Temperature coefficient a [mN/m K]	−0.1514	−0.1376
Dispersion free energy contribution $({\gamma }_{LV}^{D})$ [mN/m]	19.9	47.4
Polar free energy contribution $({\gamma }_{LV}^{P})$ [mN/m]	52.2	2.6

Surface tension, density and dynamic viscosity are evaluated at T_amb_ = 20 °C^34^.

**Figure 2:**
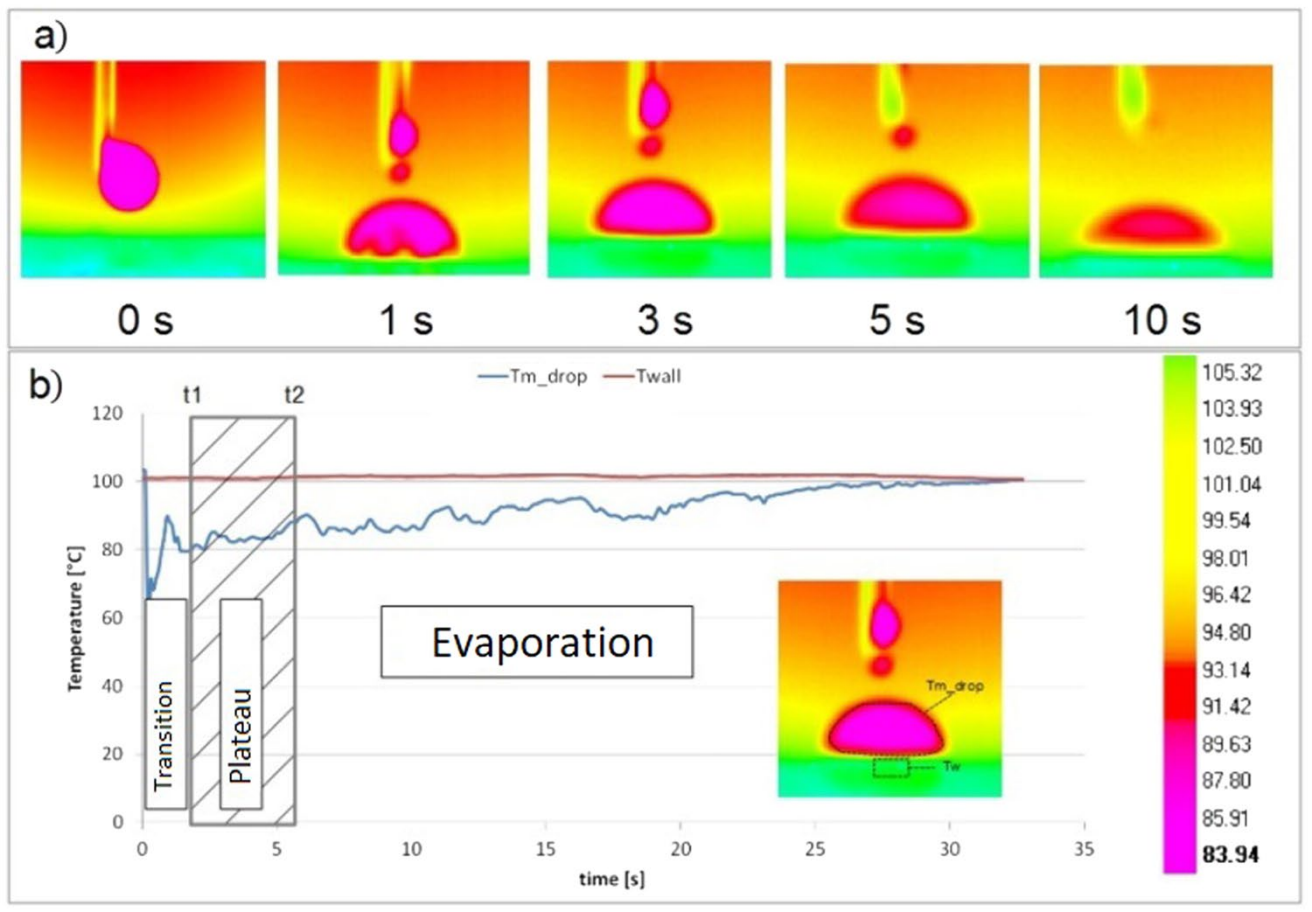
IR-camera images of a sessile water drop deposed on a hot aluminium surface. T_w_ = 100 °C. The contour plots values are set for the droplet object. The IR-camera is calibrated with emissivity of water, therefore only the temperature values of the liquid droplet are real in the images.

**Figure 3:**
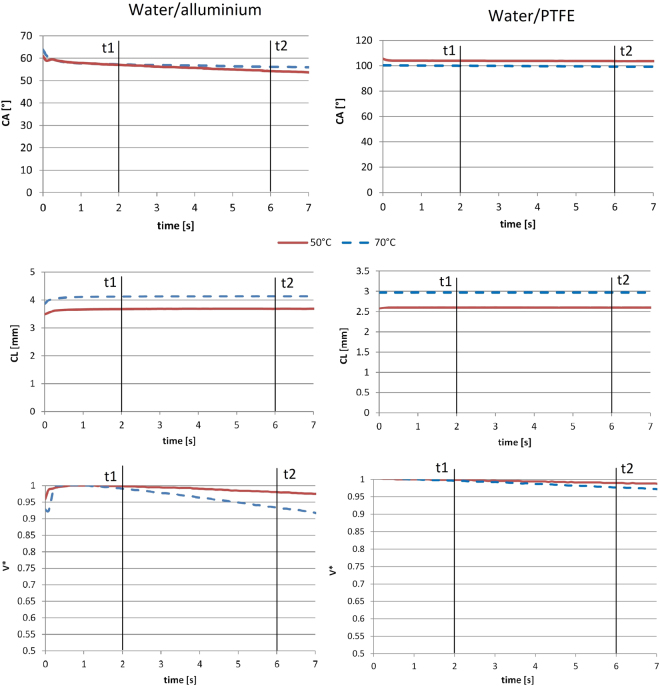
Evolution of the CA of a water droplet contact line (CL) and the non-dimensional volume (V*) of water drop on aluminium surface (on the left) and water drop on PTFE surface (on the right). Two tested surface temperatures are plotted (50 °C and 70 °C). The ECA is evaluated between t_1_ = 2 s and t_2_ = 6 s.

The evaluation of the ECA is performed in a range time called “Plateau” stage. The experimental measurement of the ECA on heated surface can be divided into 3 stages (as shown in Fig. 2b). The “Plateau” stage starts when the influence of convective motion on the value of contact angle is negligible (after the “transition” stage). The “Plateau” stage is over when the evaporation begins to change substantially the shape of the droplet (“evaporation” stage) and consequently the contact angle could be considerably influenced by evaporation^32^. The period covered by “Plateau” stage decreases with the increase of the temperature. The convective motion, as far as the evaporation rate, increases with surface temperature. Based on this assumption, the definition of the time constraints, called t1 and t2, for the “Plateau” stage can be addressed by the experiment shows in Fig. 2a. A IR-camera image of a sessile water drop deposited on a hot aluminium surface is shown. The surface temperature for this test (Tw = 100 °C) is higher than the temperature tested for the validation of the water drop ECA (20 °C < Tw < 90 °C). Using the IR-camera, it is possible to visualize the convective motion: After the gently deposition of the droplet on the surface (t = 0 s in Fig. 2a) there is indeed a generation of convective vortices in the proximity of the liquid-solid interphase (t = 1 s in Fig. 2a) due to the temperature difference between the liquid and the solid phase (that it can be reduced, but not completely removed, by heating up the liquid phase). During this initial stage (0 < t < t1), called “Transition” stage in Fig. 2b, the temperature of the droplet, initially lower than the surface temperature, increases. This convective motion, combined with the unavoidable initial drop deformation due to the deposition of the droplet on the surface, could impact the ECA measurements. After this transition stage, the droplet reaches the “Plateau” stage (in Fig. 2b). During this stage (t1 < t < t2) the ECA is constant and therefore the estimation of the ECA can be carried out in this phase. After an elapsed time (t > t2), depending on the thermal properties of the system, the drop starts to evaporate in a more vigorous mode (“Evaporation” stage in Fig. 2b). It is known that an evaporative droplet has a time-dependent contact angle^5^. The CA decreases with time due to the simultaneous reduction of the droplet volume and the initial pinning of the contact line (as describes by Stauber *et al*.^32^). The pinning of a contact line can be described by the Furmidge’s equation:

330 $${\rm{F}}={\rm{\gamma }}(\cos ({{\rm{\theta }}}_{{\rm{A}}})-\,\cos \,({{\rm{\theta }}}_{{\rm{R}}})).$$

where θ_A_ and θ_R_ are the receding and advancing contact angle. The contact line can move if the force acting on the contact line is larger than F, otherwise the pinning line doesn’t change. We define conventionally that, for our experimental conditions, the values t1 = 2 s and t2 = 6 s are the best to evaluate ECA in all the tested cases. Furthermore Fig. 3 shows the evolution of the CL (the footprint of the projected drop profile on the solid surface) and the dimensionless drop volume V* = V(t)/V(0) (V(0) is the volume of initial drop) up to t = 7 s. The CL (the footprint of the projected drop profile on the solid surface) does not show a significate change in the “Plateau” stage. For the case of water droplet on aluminium surface at Tw = 70 °C (that is the tested case with the highest evaporation rate) the dimensionless drop volume decreases less than 6%. Indeed on the aluminium surface (Fig. 3a) with T_w_ = 50° and T_w_ = 70 °C, the CA changes up to 3°, while on the PTFE (Fig. 3c) the variation of the CA is negligible. This confirms the negligible effect of the evaporation rate in the range 2 s < t < 6 s for on evaluation of ECA for our tests. During the “evaporation” stage, the evaporation is influenced by the DCA^5,32,33^.

We define here the “equilibrium contact angle” $({{\rm{\theta }}}_{ECA})$ as the average of the contact angles $({{\rm{\theta }}}_{CA})$ during the “Plateau” stage, between two times t_1_ and t_2_:

3.31 $${\theta }_{ECA}=\frac{{\int }_{t1}^{t2}{\theta }_{CA}(t)}{t2-t1}$$

## Experimental Results

**Figure 4:**
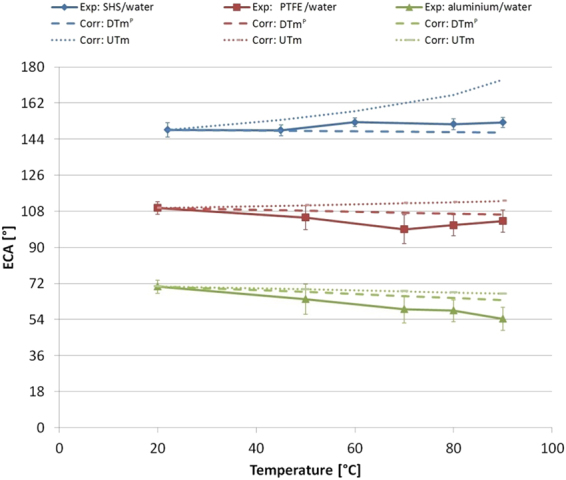
Evaluation of the equilibrium contact angle at different temperatures for a water droplet on SHS (blue) Teflon (red) aluminium (green). The temperature range is 20 °C < T_w_ < 90 °C. The experimental values are compared respectively with the Decreasing Trend Model (DTm^P^, dashed line) and the Unsymmetrical Trend Model (UTm, dotted line). The parameters of the model are set in order to have the same ECA at 20 °C.

In Fig. 4 experimental results of ECA are presented for a water drop on aluminium, PTFE and SHS. A clear decrease appears for the Hydrophilic aluminium sample (from 70.3° to 58.5°). The standard deviation in this measurement is between 5° and 7°. Also on the Hydrophobic PTFE sample a slight decrease appears (from 109.5° to 101.0°). However, the uncertainty on the calculation of equilibrium contact angles (a standard deviation of 7° is reached at higher temperatures) does not allow to conclude that the ECA varies with temperature in this case. On SHS the equilibrium contact angle is almost constant (from 148.6° to 151.6°). The trends of the ECA for these three cases are compared with the estimates from the two model DTm^P^ and UTm. The DTm^P^ and UTm show a good agreement with the experimental results for aluminium and PTFE. However, for the SHS case, with high equilibrium contact angle, the UTm predicts an increase of the equilibrium contact angle, in contradiction with the experimental data and against DTm^P^ prediction.

**Figure 5:**
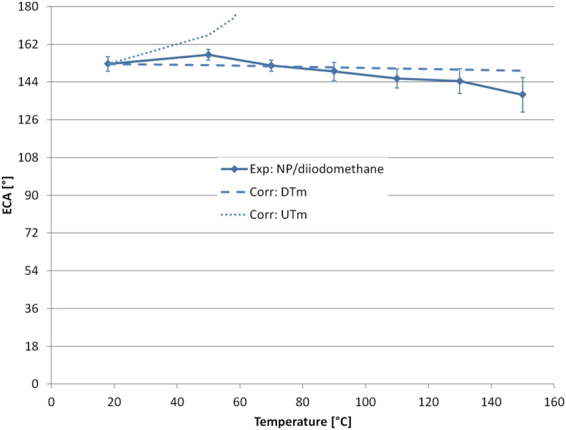
Evaluation of the ECA at different temperatures for a diiodomethane droplet on NP surface. The temperature range is 20 °C < T_w_ < 150 °C. The experimental values are compared respectively with the decreasing trend model (DTm, dashed line) and the unsymmetrical trend model (UTm, dotted line). The parameters of the model are set in order to have the same ECA at 20 °C.

Another surface (called NP) which shows a high ECA for diiodomethane is used as a further check. Figure 5 shows the comparison between the experimental measurement of ECA for diiodomethane and the two models. The prediction of DTm seems in better agreement with experimental data than UTm. The experimental data shows a decrease of the ECA (from 152.5° to 137.8°). The UTm diverges and is not able to capture the ECA value for T_w_ > 60 °C.

## Conclusions

The effect of surface temperature on equilibrium contact angle for a weakly evaporating sessile droplet sitting on a surface with different wettabilities is investigated. The “equilibrium contact angle” at a temperature higher than the standard ambient temperature is an important physical property, and an essential boundary condition in numerical simulations of phase-change phenomena and diabatic interface dynamics. However, there is some discrepancies in the literature about the value of the equilibrium contact angle with varying system temperature. Two theoretical models to predict the trend of the equilibrium contact angle (ECA) with temperatures are here proposed. The Decreasing Trend Model, with two formulations for apolar (DTm) and polar (DTm^P^) liquids, predicts a decrease of the ECA with temperature for both Hydrophilic and Hydrophobic surfaces. Instead the Unsymmetrical Trend Model (UTm) predicts a decrease of the ECA with temperature for Hydrophilic and an increase of ECA with temperature for Hydrophobic surfaces. The UTm is in contradiction with a thermodynamic approach base on the second law principle, which shows that for a homothermous system in equilibrium, an increase of the temperature cannot induce an increase of the ECA. However, since in many experiments the three phases are not really at the same temperature, the Unsymmetrical Trend Model can be still considered for the comparison with experiments. Since a sessile droplet has a time-dependent contact angle due to the droplet evaporation, a method to evaluate the ECA in function of the wall temperature is presented considering the average of the measured contact angle in a suitable time interval related to the evaporation stages and called “Plateau stage”. The ECA is here recorded for sessile droplets at ambient pressure (1 bar) on aluminium and PTFE surfaces with different wettabilities. Two fluids are used here: water and diiodomethane. For a water sessile drop, it is observed that a clear decrease of the ECA occurs for the Hydrophilic aluminium sample (from 70.3° to 58.5°). Also on the Hydrophobic PTFE sample a slight decrease appears (from 109.5° to 101.0°). However, the uncertainty on the estimate of ECA (a standard deviation up to 7° hits at high temperatures) does not allow to precisely conclude that the ECA varies with the surface temperature in this case. Instead on the SHS it is quite clear that the ECA is almost constant (from 148.6° to 151.6°). In conclusion, the DTM and UTM show a good agreement with the experimental results in the cases of aluminium/water and PTFE/water. The trends of the ECA for these two cases are compared with the estimates from the two model DTm and UTm. However, in the last two cases, SHS/water and NP/diiodomethane, UTM model predicts an increasing of the ECA in contrast with the experimental results which show a constant trend of ECA with temperature. This trend is better captured by DTM model, for both tested liquid (water and diiodomethane). In conclusion DTM approach is in better agreement with all the experimental data presented in this work.
